# Vergence Eye Movements: From Basic Science to Clinical Application - Foreword to the Special Issue

**DOI:** 10.16910/jemr.12.4.0

**Published:** 2020-03-11

**Authors:** Wolfgang Jaschinski, Rudolf Groner

**Affiliations:** Leibniz-Institut für Arbeitsforschung an der TU Dortmund; University of Bern and SciAns GmbH, Iffwil, Switzerland

**Keywords:** eye movements, Vergence, vergent eye movements, heterophoria, NPC, disparity, binocular fusion

## Abstract

The abstract book of the last European Conference on Eye Movements [1] lists abstracts of 373 presentations, but less than five percent investigate vergence eye movements, i.e. the coordination of the right and left eye. Why then a special issue on this neglected issue? Human vision under natural conditions involves both eyes in coordination controlled by interacting processes subsumed under the concept of vergence.. Further, vergence is important for people in their daily lives since disorders of vergence can have serious consequences: ophthalmologists deal with squinting patients on the basis of heterophoria and heterotropia testing, eye strain or visual complaints can be related to impaired vergence dynamic or less accurate static vergence, remediation by optometrist includes vergence training or prism eye glasses, etc.

What are the reasons why processes of vergence are underestimated in our scientific community? The main reason seems to be the fact that the two eyes must be analysed separately with high precision. The differences between the measured two eye positions are typically relatively small and often at the limit of the recording systems. The question arises: Are the difference in the measurements due to noise, to error, or due to physiological processes? A further difficulty arises from the individual differences between observers. Thus, a vergence researcher has a rather difficult job in critically evaluating the eye tracker technology and taking into account different scientific areas like ophthalmology, optometry, psychology, and using adequate statistical analyses. This kind of research requires a multidisciplinary perspective.

In this special issue, three studies concentrated on the methodology of measuring vergence. An established clinical method is the prism cover test, which measures the heterophoria, i.e. the misalignment of the visual axes under monocular viewing conditions compared to binocular fixation. Paulus, Straube amp; Eggert [2] developed an automated alternating cover test based on a combination of video-oculography and shutter glasses which minimizes stimulus noise and has a defined measurement noise. The total variance of the measurement is composed of components related to the observer, to the size of the heterophoria and to the availability of sensory vergence cues. Paulus et al. [2] examined these factors and found that a major component of the within-subject variance of the manual prism cover test is due to the variability in the manifest heterophoria of the tested persons.

Wang, Holmqvist, amp; Alexa [3] define a point of interest in binocular viewing, which is the intersection point of the two lines of sight in three-dimensional space, or – more precisely - the point closest to the two lines of sight. By means of theoretical simulations compared to empirical recordings they demonstrated a bias of the vergence distance depending on the noise of the tracked eye position. The authors propose mathematical models of calibration as part of the analysis of the experimental data.

Yaramothu, Jaswal amp; Alvarez [4] measured vergence velocity and latency for step responses and found that eccentric circles with 6° eccentricity resulted in a faster response latency than a cross at central fixation. Their results have implications for the stimulus design in a variety of applications ranging from virtual reality to interventions in vision therapy.

Vergence operates well within a limited physiological range; but when the limit of fusion is reached, single vision is lost and double vision occurs. McGinnis, I., Tierney, R., Mansell, J., amp; Phillips, J. [5] measured the clinically established convergence fusion break point (near point of convergence, NPC) by shifting a target towards the eyes in three different velocities and varied the verbal instruction (“double” versus “blurry”). The statistical analysis resulted in significant differences in NPC for the two variables target speed and verbal instruction. A consequence of this study will be that the experimental conditions for examinations and research on NPC must be standardized with respect to the experimental variables investigated in this study.

Dostalek, Hejda, Fliegel, Duchackova, Dusek, Hozman, Lukes amp; Autrata, R. [6] investigated the fusion break point at a fixed test distance, but reduced the quality of the image in one eye by different modes (luminance contrast, higher-spatial frequency content, or luminance contrast plus higher-spatial frequency content). These modes had a certain influence, but the largest effect was the one of vergence demand, i.e. the absolute disparity of the two images. The authors argue that the image's details (i.e. higher-spatial frequency content) protect binocular fusion from disruption under the lowest vergence demand.

The dynamics of vergence responses to step stimuli includes two components, a high velocity fusion initiating component followed by a slower component that may mediate sustained fusion. The slow fusionsustaining component was analysed by Semmlow and Alvarez [7]. This component was modelled by the authors as a feedback control system consisting of a time delay and an integral/derivative controller. The fast fusion-initiating component was explored by Scheiman, Yaramothu, amp; Alvarez [8] by means of analysing the ratio of the velocity divided by the response amplitude. For convergent step stimuli, this ratio was affected by a vergence/accommodation training therapy. The study of Poffa and Joos [9] used a traditional clinical method referred to as vergence facility: the examiner induces vergence responses by applying prisms and counts the number vergence movements per minute. This clinical measure was found to be related to fixations disparity, i.e. the static vergence error measured with an eye tracker.

Comparing clinical test results with eye tracker recordings were included in the two studies which took also into account individual differences. Schroth, Joos, Alshuth amp; Jaschinski [10] used a clinical nonius method for measuring the amount of the prism eye glass which is required to correct a fixation disparity (vergence error); this prism power was able to predict the prism-induced change in fixation disparity recorded with an eye tracker. Jainta and Joss [11] tested the largest sample of subjects in this issue (n= 94) which allowed demonstrating the influence of the individual heterophoria on the binocular advantage, i.e. the extent to which during reading the fixation of a word is shorter in binocular than in monocular reading. The eye tracker measure of the heterophoria achieved superior results compared with subjective clinical measurements.

The academic background of the present authors illustrates that vergence research is covered by different scientific disciplines including computer engineering, physics, optometry, ophthalmology and psychology. This has the advantage that vergence research benefits from the different approaches of these disciplines, given that a common language and mutual understanding is achieved. A common basis for such a multidisciplinary research could be the seminal book of Ian Howard [12].

This first special issue on vergence eye movements should give an overview of ongoing research in a relatively small scientific community and might motivate more relevant and multidisciplinary research, to be published in regular issues of the Journal of Eye Movement Research.
